# Tracking stress via the computer mouse? Promises and challenges of a potential behavioral stress marker

**DOI:** 10.3758/s13428-021-01568-8

**Published:** 2021-04-05

**Authors:** Paul Freihaut, Anja S. Göritz, Christoph Rockstroh, Johannes Blum

**Affiliations:** Department of Occupational and Consumer Psychology, Albert-Ludwigs-Universität Freiburg, Engelbergerstr. 41, 79106 Freiburg, Germany

**Keywords:** Computer mouse, Tracking, Behavior, Stress, Measurement, Machine learning

## Abstract

Computer mouse tracking offers a simple and cost-efficient way to gather continuous behavioral data and has mostly been utilized in psychological science to study cognitive processes. The present study extends the potential applicability of computer mouse tracking and investigates the feasibility of using computer mouse tracking for stress measurement. Drawing on first empirical results and theoretical considerations, we hypothesized that stress affects sensorimotor processes involved in mouse usage. To explore the relationship between stress and computer mouse usage, we conducted a between-participant field experiment in which *N* = 994 participants worked on four mouse tasks in a high-stress or low-stress condition. In the manipulation check, participants reported different stress levels between the two conditions. However, frequentist and machine learning data analysis approaches did not reveal a clear and systematic relationship between mouse usage and stress. These findings challenge the feasibility of using straightforward computer mouse tracking for generalized stress measurement.

The advancing availability of sensors that capture dynamic and real-time physiological or behavioral data in everyday life offers great potential for psychological science (Bauer et al., 2020; Salas et al., 2017). In the advent of technologies such as smartwatches and fitness trackers, the computer mouse as a long-standing, ubiquitous sensor has largely been overlooked, although it captures dynamic data about human behavior with high temporal resolution (Hehman et al., 2015). In recent years, researchers have successfully started to utilize the potential of the computer mouse to study cognitive processes (Freeman, 2018). The present work focuses on the feasibility of using the computer mouse for stress measurement. Apart from the intuitive assumption that affective states such as stress or fatigue influence the way one uses the computer mouse, empirical research on the topic is sparse. We try to advance the field by (1) summarizing the state of the art, (2) reporting empirical evidence from a large-scale experiment, and (3) providing our material and code as guidance on how one might work with mouse usage data in the context of affect measurement (10.5281/zenodo.4004776).

## Theory

### Affective computing and psychological science

The research field of affective computing has been the main driver for sensor-based affective state measurement. Affective computing refers to the interdisciplinary study and development of computer systems that can recognize, interpret, process, and simulate human affects (Picard, 2014). Behavioral or physiological data captured by sensors such as a camera or an electrocardiogram (ECG) are the basis for an “affective computer” (for a review see Calvo & D’Mello, 2010). Similar to affect recognition in humans, the data represent cues about the affective state of the interaction partner, which the computer must “learn” to interpret and then react to appropriately. From a psychological point of view, affective computing not only represents a fascinating application of psychological knowledge; the developments in affective computing also offer potential for psychological science itself. Particularly, sensors that continuously record data about behavioral actions and cognitive, physiological, or affective states are powerful tools for the fine-grained and dynamic study of psychological phenomena (Adjerid & Kelley, 2018; Salas et al., 2017). In such a way, both research areas strengthen each other reciprocally. Affective computing provides the methodology to gather and analyze data about affective states, while psychological research provides the theoretical foundation about affective states and therefore fosters a more precise applicability of the methods.

This synergy is especially noticeable in stress research. Stress is an omnipresent characteristic of contemporary life in western societies, with an increasing prevalence (American Psychological Association, 2018). Many individuals in today's world are confronted with a high number of emotional and cognitive demands (Grönlund, 2007), whose accumulation can lead to chronic stress if the individual is not able to adequately cope with or recover from the demanding episodes (McEwen & Seeman, 2003). Chronic stress is considered a threat to physical and mental health, imposing immense costs on individuals and society at large (Hassard et al., 2018; Scott et al., 2018). Consequently, the development of novel countermeasures is of obvious importance. Using sensor data to unobtrusively monitor behavioral or physiological processes associated with the stress reaction represents a potentially innovative diagnostic tool for stress measurement that allows delivering preemptive and just-in-time interventions (Alberdi et al., 2016). As stress is a complex psychophysiological phenomenon (De Kloet et al., 2005; McEwen, 2000), capturing dynamic and fine-grained sensor data related to stress offers an exciting methodological avenue for its deeper understanding and a more precise disclosure about the relationship between acute stressful episodes and chronic stress (Bliese et al., 2017). The need for a better comprehension of stress also shows in the lack of a uniform definition (McEwen, 2000). In the present paper, we define stress as a state of negative tension (i.e., strain reaction) resulting from a situation that is perceived as threatening and expected to exceed one’s coping resources (cf. Zapf & Semmer, 2004).

### The computer mouse as a stress detector

Computer mouse tracking represents a behavioral sensor-based stress measurement approach that has largely been overlooked in affective computing research, although it offers a potentially exciting addition to established stress measurement approaches (for reviews on sensor-based stress measurement approaches in affective computing, see Alberdi et al., 2016; Can et al., 2019). The major advantage of the computer mouse is that it represents a ubiquitous sensor that is integrated in everyday life and allows for continuous data collection without the need for sophisticated equipment and without requiring the user to change their customary behavior or habits. For this reason, the computer mouse is an ideal candidate for an objective, cheap, convenient, and unobtrusive measurement instrument in settings where computers are frequently used (e.g., the office or research laboratories).

Drawing on these benefits, in cognitive science, computer mouse tracking has become a popular research method in a wide range of applications (for reviews see Freeman, 2018; Stillman et al., 2018). Its promise is provision of fine-grained temporal data, which helps to reveal the dynamics and microstructure of cognitive processes in real-time (Freeman, 2018). In a typical experimental setup, participants are presented with several options on a computer screen and have to use the computer mouse to navigate to and click on one option based on a given rule. The resulting mouse trajectories during the task carry information about the cognitive processes involved in the decision-making process (Freeman, 2018). For example, in self-control research, the mouse trajectories of participants with higher levels of self-control revealed a reduced tendency to navigate towards an unhealthy food option when having to choose a healthy over an unhealthy food option (Stillman et al., 2017). Such findings let researchers conclude that mouse trajectories reflect a continuous decision making process *(continuity of mind hypothesis*, Spivey, 2008), although reanalysis of existing mouse trajectory studies with a novel cluster analysis approach challenge some of the conclusions of previous research (Wulff et al., 2019). To this effect, mouse tracking continues to facilitate theoretical advancements in the understanding of cognitive processes. Software to apply mouse tracking in cognitive science is freely available (Freeman & Ambady, 2010; Kieslich & Henninger, 2017).

In affective computing, Zimmermann et al. first described the potential of using the computer mouse for affective state measurement in 2003. Empirical evidence on the topic, however, is sparse. Most studies are reports about pilot projects that show mixed evidence about a relationship between computer mouse usage and different affective states without a clear indication of a systematic pattern (cf. Grimes et al., 2013; Grimes & Valacich, 2015; Hernandez et al., 2014; Kaklauskas et al., 2011; Macaulay, 2004; Salmeron-Majadas et al., 2014; Zimmermann, 2008). Yamauchi and Xiao (2018) also pointed out the small sample sizes and methodological shortcomings of most studies.

There are only a few comprehensive studies. Hibbeln et al. (2017) showed that the participants’ mouse speed and traveled mouse distance were related to their self-rated (negative) valence in three separate experiments (total *N* = 271). Each experiment used a different task and design: (1) solving a puzzle after an unfair or fair intelligence test (between-groups design), (2) ordering an item in an online-shop with loading delays or without loading delays (between-groups design), and (3) using a car/computer configurator (correlative design). Yamauchi and Xiao (2018) conducted four experiments (total *N* = 897) that all used a decision-making task in which participants had to select one of two geometrical figures, which that was most similar to a third figure. In each experiment, the authors used a different emotion manipulation and/or collected different self-reported emotional states. The results revealed that mouse usage (operationalized as the distance from an ideal line and the number of directional changes) was correlated to some of the emotional states measured in each experiment, but the correlations were not entirely consistent across the experiments. In two similar experiments (total *N* = 355), Yamauchi et al. (2019) demonstrated that viewing emotional pictures affected mouse usage (measured as the distance from an ideal line and the area under the curve as spatial mouse features and the peak velocity and acceleration of the mouse as temporal features). Pimenta et al. (2016) collected ten different mouse usage features (e.g., speed, acceleration, time between two mouse clicks, distance between two mouse clicks) of 24 participants during classwork in a computer laboratory to predict the self-rated fatigue level at 81% accuracy.

Few studies focused on stress, specifically. Freihaut and Göritz (2021) conducted a within-subjects laboratory experiment (*N* = 53) and did not find systematic differences in 24 mouse usage parameters (e.g., average mouse speed, total mouse distance) during four different mouse usage tasks between a high- and low-stress condition. Sun et al. (2014) also conducted a within-subjects laboratory experiment (*N* = 49) and found differences in mouse usage operationalized as damping frequency and damping ratio (i.e., measures for muscle stiffness) between a high- and low-stress condition, although there was no difference in physiological arousal between the conditions. In another laboratory study (*N* = 18), Kowatsch et al. (2017a) showed that mouse speed and deviation from an ideal line differed between a training and test trial in a high-stress but not in a low-stress condition. The same authors (2017b) collected mouse speed as well as valence and arousal ratings of office workers (*N* = 62) during their everyday computer usage in a field study that spanned several days. There was no correlation between mouse speed, on the one hand, and valence or arousal on the other hand, but a correlation was found with a combined valence-arousal score.

In sum, the empirical evidence tentatively points towards a relationship between mouse usage and affective states, but remains blurry. To add to the uncertainty, the high number of preliminary studies without follow-up might indicate some degree of publication bias. Importantly, the presented studies used different data preprocessing steps, extracted different mouse usage features, collected the data during different tasks, and analyzed the data using different procedures. This large number of methodological degrees of freedom increases the likelihood of finding (and reporting) unreliable outcomes, especially if exploratory data analytical principles are violated (e.g., not validating the findings on an independent dataset; Kuhn & Johnson, 2013). Taken together, this stresses the importance of tying the research to a theoretical framework on the one hand, and the disclosure of the data and data analytical procedures on the other hand. To the best of our knowledge, none of the aforementioned studies disclosed their data and data analyses, and only some provided a theoretical background (cf., Hibbeln et al., 2017; Yamauchi & Xiao, 2018).

### Theory linking stress to computer mouse usage

Navigating the mouse to execute a task on the computer is a goal-directed sensorimotor action. Research on motor control suggests that the underlying processes in sensorimotor actions are complex (Gallivan et al., 2018), and some researchers argue that motor control is the main reason for the existence of the brain (Wolpert, 2011). A goal-directed reaching movement, such as steering the mouse cursor to a button and clicking on it, involves the succession of multiple processes governed by different feedback and regulation mechanisms (i.e., planning the movement, making an initial movement impulse towards the target, adjusting the movement impulse to reach the target) and requires balancing of demands for speed, accuracy, and energy costs of the resulting movement (multiple process model, see Elliott et al., 2010, 2017). Although theoretical models of motor movement have improved over time, it remains a fundamental challenge to better uncover associated sensorimotor and cognitive processes as well as their interplay (Elliott et al., 2017). A lack of theory to predict the effect of stress—being a complex psychophysiological phenomenon itself—on motor control therefore comes as no surprise.

In their theoretical reasoning, Hibbeln et al. (2017) and Yamauchi and Xiao (2018) point out the potential role of working memory and attention on the planning and execution of goal-directed actions (Gallivan et al., 2016; Mattek et al., 2016; Welsh, 2011; Xiao & Yamauchi, 2017) and affective states’ interference with them (Domínguez-Borràs & Vuilleumier, 2013; Eysenck et al., 2007). Similarly, stress is known to have a detrimental impact on cognitive functions (Arnsten, 2009) such as working memory (Oei et al., 2006; Qin et al., 2009; Schoofs et al., 2008), attentional control (Sänger et al., 2014), selective attention (Elling et al., 2011), and cognitive control (Plessow et al., 2012).

Neurological and biomechanical research, too, support the idea that affective states and especially stress influence computer mouse usage (Hibbeln et al., 2017). Exposure to stressful or emotional stimuli increased corticospinal excitability (Coelho et al., 2010), motor evoked potentials and muscle activity (Finsen et al., 2001; Laursen et al., 2002; Lundberg et al., 1994), facilitated force production (Coombes et al., 2008; Naugle et al., 2012) and influenced motor performance (Tanaka et al., 2012). Visser et al. (2004) found increased muscle activity and force extension on the computer mouse as well as changes in task performance and mouse usage behavior in high versus low mentally demanding tasks. Muscle activity has also been used for stress measurement in affective computing research (Greene et al., 2016). In their theory of stress and human motor performance, van Gemmert and van Galen (1997) argue that stress both activates the motor system and enhances neuromotor noise, which can affect task performance. They provide support for their hypothesis in multiple studies (e.g., van Galen et al., 2002; van Galen & van Huygevoort, 2000; van Gemmert & van Galen, 1997). In a review of 31 studies, Staal (2004) concluded that stress impairs motor performance, with fine motor skills being at greater risk of impairment.

In sum, both theoretical considerations and empirical findings point towards a relationship between mouse usage and stress. However, with regard to the complex underlying processes and the inconclusive evidence, it seems premature to postulate concrete hypotheses about causal relationships between stress or other affective states and specific mouse usage parameters. Similar to Yamauchi and Xiao (2018), the present study therefore follows an exploratory approach. The goal of the present study was to systematically search for meaningful empirical evidence in favor of a relationship between stress and mouse usage. Such information may then provide the starting point to theory advancement and may sharpen the understanding of the underlying processes. To explore the relationship between mouse usage and stress, we conducted a web-based experiment that included four prototypical mouse usage tasks. The online setting is a unique characteristic of the present study, with the exception of one of the three experiments by Hibbeln et al., 2017. Capturing mouse usage in the participants’ natural environment with their own hardware strengthens the study’s external validity and allows for a better judgement of the practicability of the measurement approach, while the experimental design preserves much of the study’s internal validity.

## Method

### Participants

Participants were recruited via WisoPanel, an online access panel comprising research participants with demographic characteristics that resemble the German population (Göritz, 2009; Göritz, 2014). All 14,343 panel members received an invitation by e-mail that included a link to the study. The link was opened by 1,941 participants (response rate: 15.65%) of which 1,091 completed the study in exchange for a remuneration of 1 Euro (retention rate: 56.21%). We removed 97 participants (8.89%) because they showed signs of careless responding or technical difficulties (see code for further details) resulting in a final sample of *N* = 994 (mean age = 54.4, *SD* = 13.3; 515 women, 479 men). The study’s requirements included the use of a physical computer mouse, a minimum display resolution of 950 × 600 and a modern web browser. As far as technologically possible, we checked the requirements before the start of the study, and filtered out and informed the participant in the case of a violation. The median study duration was 21 min.

### Design

The experiment had a between-subjects design and consisted of two stages. In the first stage (baseline stage), all participants had to run through four different mouse tasks once for practice and to capture a baseline. In the second stage (application stage), participants were randomly assigned to work on the practiced mouse tasks in either a high-stress (*n* = 480) or low-stress (*n* = 514) condition. The experiment was programmed as a single page web application with the JavaScript framework react.js and Firebase as a backend. The content on the web page was horizontally and vertically centered and had a fixed size.

### Stress manipulation

The aim of the stress manipulation was to create a constantly high (versus low) stress level during all mouse tasks in the high-stress (versus low-stress) condition. In line with our stress definition, we confronted participants with a situation they perceived as threatening (versus neutral) and that exceeded (versus mildly challenged) their cognitive resources: We let participants work on a hard (versus easy) stress manipulation task before each mouse task and used a threatening (versus neutral) framing.

#### Stress manipulation task

**Fig. 1:**
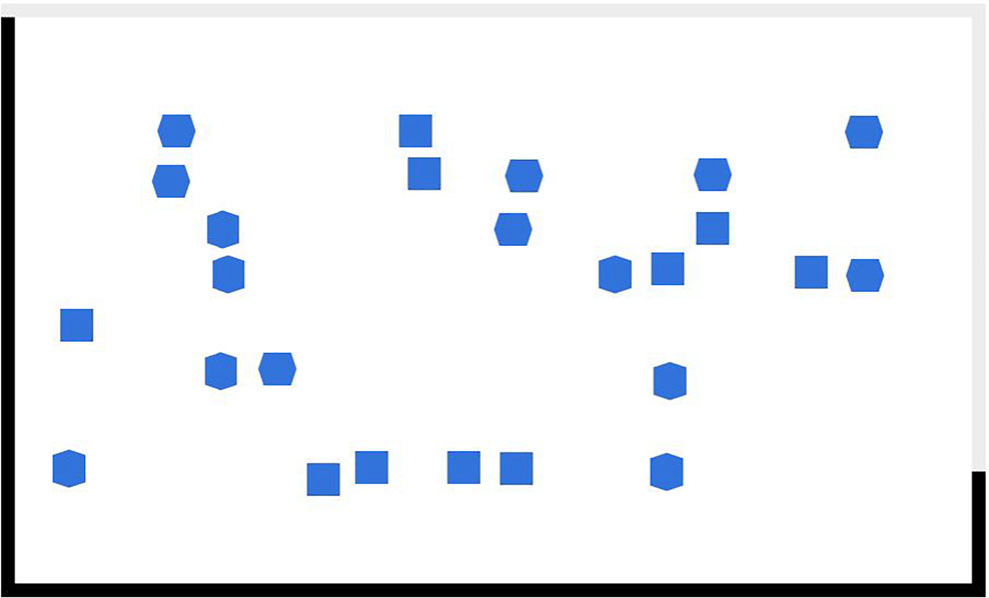
Screenshot of the counting task

We used a self-developed counting task (Fig. 1) for manipulating stress. Although there exist various stress manipulation tasks (for an overview, see Ferreira, 2019), we felt that none fully met this study’s requirements: the task must be executable in an online setting, must be amenable to be presented multiple times, must be intuitive and easy to understand, must have an adequate control condition, and must not require any mouse usage. The counting task is a standardized task in which participants are shown successive screens with a varying number of three similar geometrical shapes (i.e., square, horizontal hexagon, vertical hexagon) and are instructed to count only the squares. The stress manipulation task consisted of seven successive counting trials with a duration of 5 s each. A loading bar visualized the remaining time in each trial. There was a delay of 1,250 ms between trials and a fixation cross, which was shown for 750 ms, indicated the start of a new trial. The mouse cursor was invisible during the entire task. At the end of the task, participants had 10 s to type the number of squares they had counted during the task into an input field. The two conditions differed in the number of presented squares and distractors. In the high-stress condition, participants saw a total of 287 squares and 798 distractors. In the low-stress condition, participants saw 115 squares and 319 distractors. We chose the numbers to create a balanced task difficulty that was slightly too hard but not impossible for most participants in the high-stress condition versus easy to manage but not too trivial in the low-stress condition.

#### Framing

Besides the stress manipulation task, the conditions differed in the announced purpose. In the high-stress condition, participants were told that the ensuing sequence of tasks amounts to a performance test that measures some facet of intelligence (i.e., threat framing). In the low-stress condition, participants were told that the ensuing sequence of tasks is an application of the mouse tasks they had already practiced at the beginning and that would teach them skills for working on computerized tasks more generally (neutral framing). The framing was to add a social-evaluative element to the cognitive load element of the counting task as social-evaluative threat has shown to elicit a strong psychobiological stress response in the laboratory (Dickerson & Kemeny, 2004). In both conditions, participants were promised feedback on their performance and were asked to work on all tasks as fast and as accurately as possible. The word count in the framing was identical in both conditions.

### Mouse tasks

We created four mouse tasks to capture different prototypical goal-directed mouse usage actions (Sun et al., 2014). All tasks were identical in the baseline stage and application stage. The mouse tasks were also identical in the high-stress and low-stress condition to prevent that task-related differences confound potential effects of stress on mouse usage.

#### Point-and-click task

**Fig. 2:**
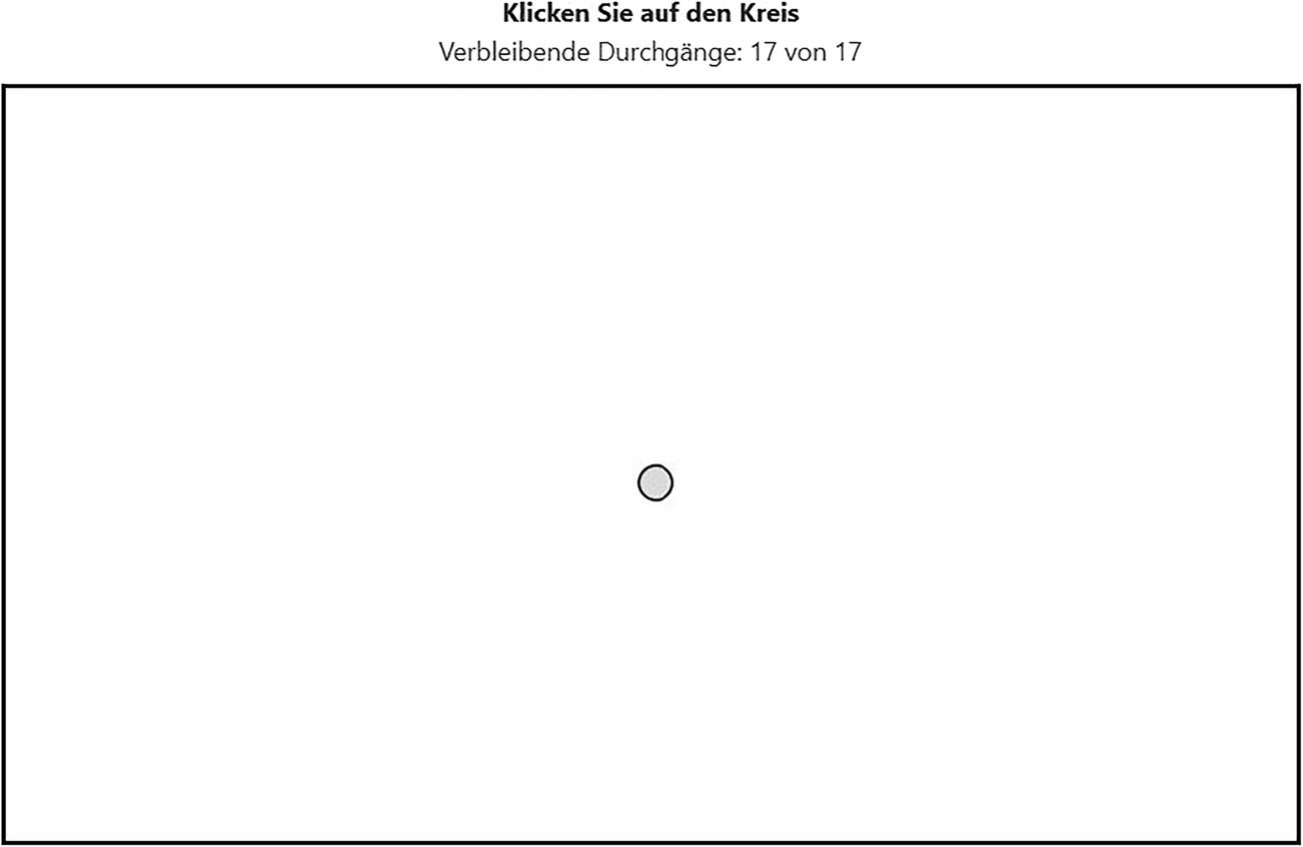
Screenshot of the point-and-click task. Translation of instructions above the black-framed playing field: Click on the circle (in bold); remaining trials: 17 out of 17

In the point-and-click tasks (Fig. 2), participants had to click on 17 circles, which successively appeared on different positions inside a playing field. A counter above the playing field showed the remaining number of circles to click on.

#### Drag-and-drop task

**Fig. 3:**
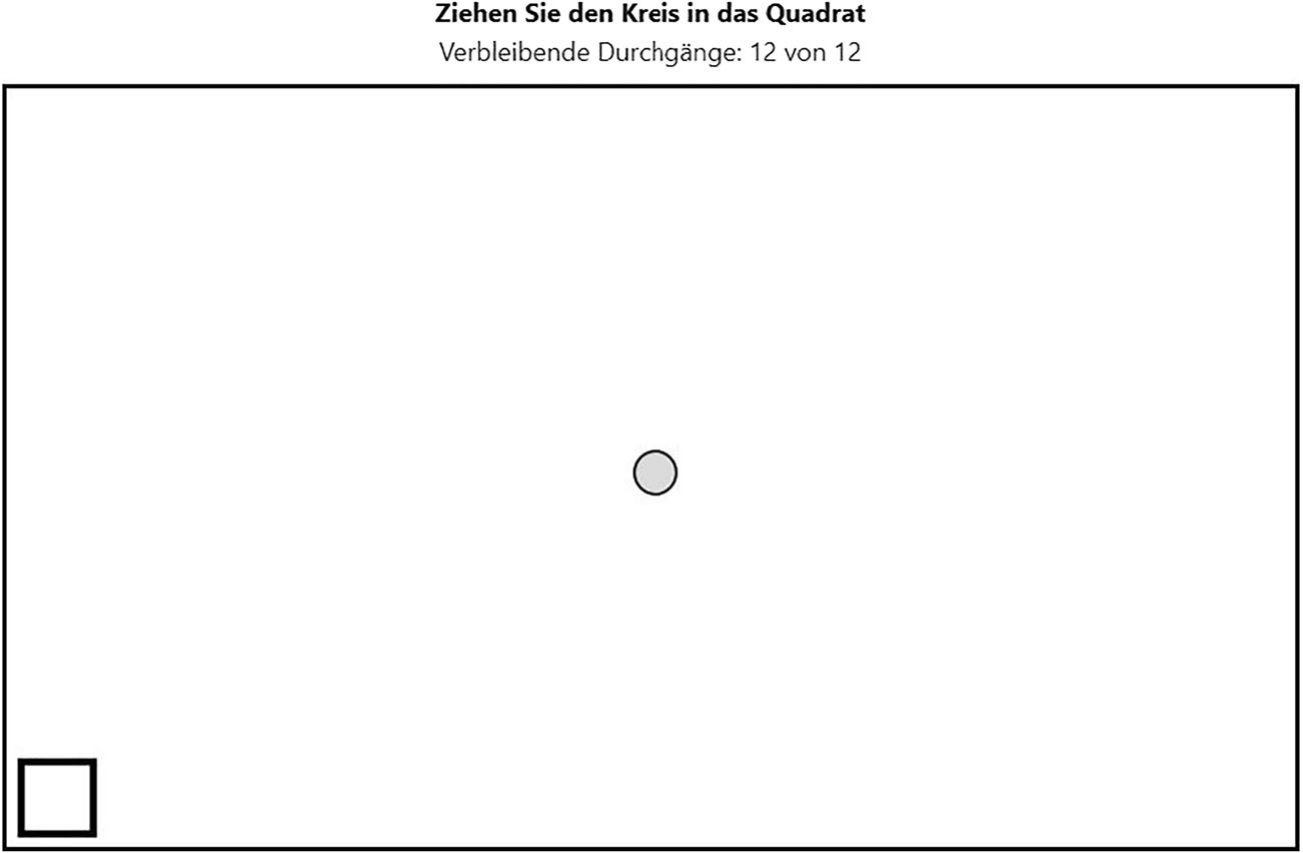
Screenshot of the drag-and-drop task. Translation of instructions above the black-framed playing field: Drag the circle into the square (in bold); remaining trials: 12 out of 12

In the drag-and-drop task (Fig. 3), participants had to drag and drop 12 circles from the center of the playing field into a squared target box that successively appeared in one of the corners of the playing field. If the circle was dropped outside of the target box or dragged outside of the playing field, its position was reset to the center. The target box’s color indicated whether the circle was inside the target box and ready to be dropped. A counter above the playing field showed the remaining number of circles to drag and drop.

#### Slider task

**Fig. 4:**
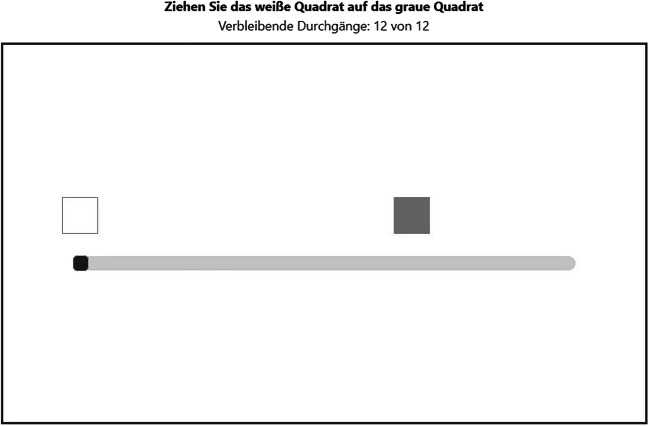
Screenshot of the slider task. Translation of instructions above the black-framed playing field: Drag the white square onto the gray square (in bold); remaining trials: 12 out of 12

In the slider task (Fig. 4), participants had to move the handle of a horizontal slider in such a way that a white square, which moved along with the slider, fully covered an equal-sized gray square that successively appeared on different positions on a horizontal axis. The task had 12 trials. After each trial, the slider and the white square were reset to the starting position. A counter above the playing field showed the remaining number of slides.

#### Follow-the-circle task

**Fig. 5:**
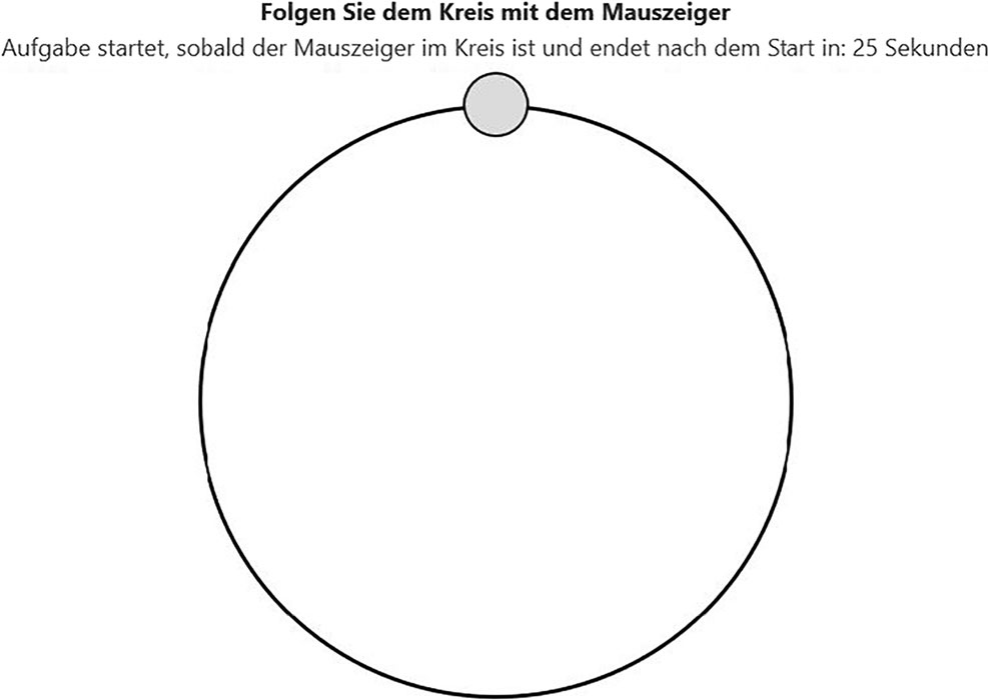
Screenshot of the follow-the-circle task. Translation of instructions: Follow the circle with the mouse cursor (in bold); Task starts as soon as the mouse cursor moves inside the circle and ends after: 25 s

In the follow-the-circle task (Fig. 5), participants had to keep their mouse cursor inside a circle that underwent a radial movement for 25 s at a constant velocity. The task is similar to the Pursuit Rotor Task (Adams, 1952), which is a task to measure motor coordination. The circle started to move when the participant moved the mouse cursor inside it. The circle’s color indicated whether the mouse cursor was inside or outside of it. A countdown above the playing field showed the remaining time.

### Measures

Computer mouse usage was captured on the client-side via the web application. The data was collected in an event-based manner, that is, a data point was created every time a mouse event (i.e., positional change or click) occurred. Because of the client-sided data collection, the maximum sampling frequency of continuous mouse movement differed between participants in a range between 20 Hz and 200 Hz (md = 60 Hz). Each data point consisted of the name of the mouse event, the cursor’s x/y position on the screen, a timestamp, and additional task-specific information (e.g., the number of circles clicked so far in the point-and-click task).

Participants’ stress levels were measured via self-report. After each mouse task in the baseline stage and application stage, participants rated how they felt in terms of valence (from 0 = *positive* to 4 = *negative*) and arousal (from 0 = *calm* to 4 = *excited*) on the Self-Assessment-Manikin (SAM, Bradley & Lang, 1994). Furthermore, at the end of both the baseline stage and application stage, participants rated their affective state in more detail on Version B of the German Multidimensional Mood Questionnaire (Mehrdimensionaler Befindlichkeitsbogen [MDBF]; Steyer et al., 1997). The questionnaire consists of twelve items about emotional states (e.g., “I'm feeling calm”), which are rated on a 5-point scale (0 = *not at all*, 4 = *very much*) and summarized into the three bipolar subscales: good mood versus bad mood (Cronbach’s α in the present study ≥ 0.87), rest versus unrest (Cronbach’s α ≥ 0.84), and alertness versus tiredness (Cronbach’s α ≥ .90). We appended one item asking directly about the stress level (“I'm feeling stressed”) and one item asking about nostalgia (“I'm feeling nostalgic”). The nostalgia item was used to test the specificity of the stress manipulation, as the stress manipulation should affect stress-related affective states but not the feeling of nostalgia.

### Procedure

**Fig. 6:**
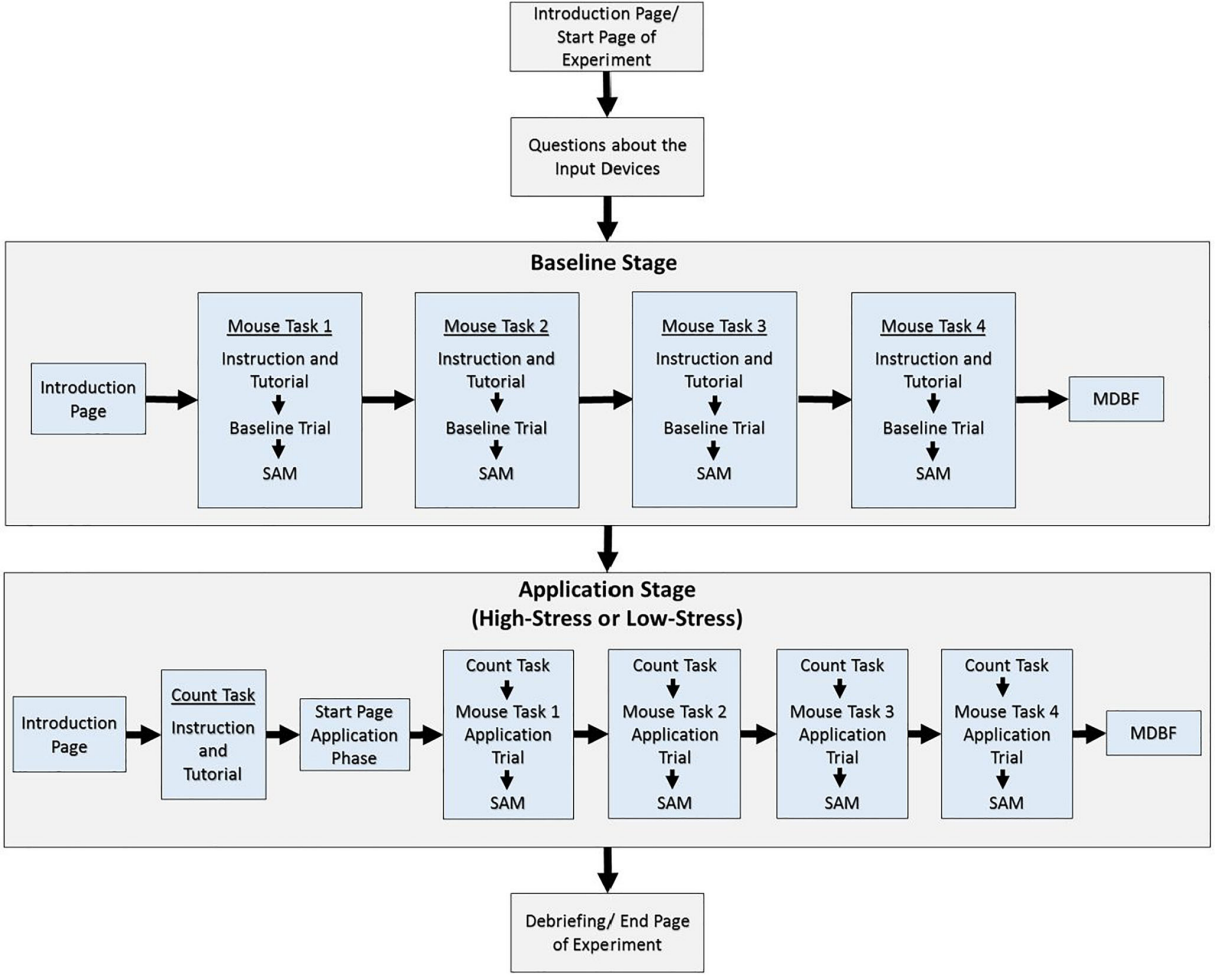
Flowchart of the experimental procedure

On opening the study’s link, participants first saw an introduction page with study information. Regarding the study’s purpose, participants were informed that the study was about the processing of different computerized tasks. Participants had to give consent before starting the experiment. In the first part of the experiment, participants had to self-check whether they were using a computer mouse. If they answered no, they were re-informed about the participation requirements and asked to restart the study with a computer mouse. If they answered yes, they had to indicate whether they were using the computer mouse with their right or left hand and whether they were using a built-in keyboard on a laptop. Next in the experiment was the baseline stage. Here, participants were introduced to and completed a baseline trial of each mouse task. The task instructions included written information and a tutorial version of the task. The task order was randomized. After each task, participants rated their current affect in terms of valence and arousal on the SAM. At the end of the baseline stage, participants filled out the MDBF plus stress and nostalgia item. Next was the application stage. It started with an introduction to the counting task, which included written information and a tutorial version of the counting task. Afterwards, participants worked on triples of the counting task, followed by a mouse task, and concluded by the SAM until they had completed all tasks. The order of the mouse tasks in the application stage was the same as in the baseline stage. At the end of the application stage, participants filled out the MDBF plus the stress and nostalgia item again. The study ended with a debriefing about the study purpose and stress manipulation. Participants were also shown their accumulated answer of all counting tasks in comparison to the accumulated solution of all counting tasks (see Fig. 6 for a flowchart of the experiment).

### Mouse data preprocessing

The preparation of the mouse data for analysis included multiple processing steps per mouse task (see code for detailed information): (1) We selected all data points for the respective task. (2) We removed artifacts from the task data, which were consecutive mouse movement data points with either the same timestamp or identical *x*- and *y*-coordinates. (3) We visually inspected the data for potential tracking difficulties. (4) We removed participants from the task if they showed signs of tracking difficulties or if their task duration was more than three times greater than the median task duration. (5) We linearly interpolated the mouse movement data into equally long intervals of 15 ms. (6) We computed a range of mouse usage features. The features were selected to adequately represent mouse usage behavior during each task and can be divided into 8 temporal features, 5 spatial features, and 4 task-specific features (Table 1 for an overview of features).

**Table 1:** Description of mouse usage features

Feature name	Feature description	Calculated for mouse task
Temporal features		
Task time	Time difference between the last and first data point on the task page in seconds	point-and-click task, drag-and-drop task, slider task, follow-the-circle task
Working time	Time difference between the last data point on the task page and the first data point when participants started working on the task in seconds (e.g., the click on the first circle in the point-and-click task)	point-and-click task, drag-and-drop task, slider task, follow-the-circle task
Average mouse speed	Average speed of the mouse cursor during the task. Mean of ∆Euclidean distance/∆time between consecutive mouse movement data points in pixels per second	point-and-click task, drag-and-drop task, slider task, follow-the-circle task
Standard deviation of mouse speed	Standard deviation of mouse cursor speed during the task. Std. of ∆Euclidean distance/∆time between consecutive mouse movement data points in pixels per second	point-and-click task, drag-and-drop task, slider task, follow-the-circle task
Average positive mouse acceleration	Average positive acceleration of the mouse cursor during the task. Mean of ∆+speed/∆time between consecutive mouse speed data points in pixels per s^2^	point-and-click task, drag-and-drop task, slider task, follow-the-circle task
Standard deviation of positive mouse acceleration	Standard deviation of positive acceleration of the mouse cursor during the task. Std of ∆+speed/∆time between consecutive mouse speed data points in pixels per s^2^	point-and-click task, drag-and-drop task, slider task, follow-the-circle task
Average negative mouse acceleration	Average negative acceleration of the mouse cursor during the task. Mean of ∆-speed/∆time between consecutive mouse speed data points in pixels per s^2^	point-and-click task, drag-and-drop task, slider task, follow-the-circle task
Standard deviation of negative mouse acceleration	Standard deviation of negative acceleration of the mouse cursor during the task. Std of ∆-speed/∆time between consecutive mouse speed data points in pixels per s^2^	point-and-click task, drag-and-drop task, slider task, follow-the-circle task
Spatial features		
Total mouse distance	Total mouse distance traveled during the task. Sum of Euclidean distances between consecutive mouse movement data points in pixels	point-and-click task, drag-and-drop task, slider task, follow-the-circle task
Average mouse angle	Average angle of the mouse movement during the task. Mean of angles between consecutive mouse movement vectors (three consecutive mouse movement data points) in degrees	point-and-click task, drag-and-drop task, slider task, follow-the-circle task
Standard deviation of mouse angle	Standard deviation of angles of the mouse movement during the task. Std of angles between consecutive mouse movement vectors (three consecutive mouse movement data points) in degrees	point-and-click task, drag-and-drop task, slider task, follow-the-circle task
Changes in *x*-direction	Number of directional changes in the *x*-direction (horizontal movement)	point-and-click task, drag-and-drop task, slider task, follow-the-circle task
Changes in *y*-direction	Number of directional changes in the *y*-direction (vertical movement)	point-and-click task, drag-and-drop task, slider task, follow-the-circle task
Task-specific features		
Total deviation from ideal line	Total deviation in the movement from an ideal line representing the straight connection between start and end positions of a trial. Sum of the deviations from an ideal line of every movement data point	point-and-click task, drag-and-drop task
Mean deviation from ideal line	Average deviation in the movement from an ideal line representing the straight connection between start and end positions of a trial. Mean of deviations from an ideal line of every movement data point	point-and-click task, drag-and-drop task
Standard deviation of deviation from ideal line	Standard deviation of the deviation of the movement from an ideal line representing the straight connection between start and end positions of a trial. Std of deviations from an ideal line of every movement data point	point-and-click task, drag-and-drop task
In circle ratio	Ratio of the time the mouse cursor was inside the task circle in the follow-the-circle task to the time the mouse cursor was outside of the task circle	follow-the-circle task

## Results

First, we tested the success of the stress manipulation. Next, we used two statistical approaches to explore the relationship between stress and mouse usage: (1) frequentist analysis to compare the mouse usage features between the conditions individually and (2) machine learning to test globally whether there is a systematic pattern in mouse usage related to stress.

### Manipulation check

We tested the success of the stress manipulation, both as an overall difference between the high-stress and low-stress condition on the subscales of the MDBF and the perceived stress and nostalgia item, as well as the SAM difference between the high-stress and low-stress condition in each mouse task. For each dependent variable, we conducted a mixed analysis of variance (ANOVA) with Condition (high-stress vs. low-stress) as the between-subjects factor and Stage (baseline stage vs. application stage) as the within-subjects factor. The analyses were carried out using the *pingouin* package in Python (Version 0.3.3; Vallat, 2018).

As regards the difference between the conditions, we found significant interaction effects between Condition and Stage on the good mood versus bad mood MDBF subscale, *F*(1, 992) = 12.28, *p* < .001, η^2^_part_ = 0.012, the rest versus unrest subscale, *F*(1, 992) = 13.35, *p* < .001, η^2^_part_ = 0.013, the alertness versus tiredness subscale, *F*(1, 992) = 6.20, *p* = .013, η^2^_part_ = 0.006, and for perceived stress, *F*(1, 992) = 15.22, *p* < .001, η^2^_part_ = 0.015, but not on nostalgia, *F*(1, 992) = 1.17, *p* = .279, η^2^_part_ = 0.001. Descriptively, the changes in scores are in line with the aim of the stress manipulation. In the high-stress condition, there is a stronger increase in bad mood (Δ_high-stress_ = −0.14, Δ_low-stress_ = −0.04), unrest (Δ_high-stress_ = −0.26, Δ_low-stress_ = −0.13), and stress (Δ_high-stress_ = 0.33, Δ_low-stress_ = 0.13) from the baseline stage to the application stage as compared to the low-stress condition. As regards alertness versus tiredness, in the high-stress condition, participants reported feeling more tired in the application stage as compared to the baseline stage (Δ_high-stress_ = −0.05), while in the low-stress condition, participants reported feeling more awake in the application stage (Δ_low-stress_ = 0.03). Post hoc comparisons revealed no significant between-group differences for any variable in the baseline stage, but in the application stage, participants in the high-stress condition felt worse than those in the low-stress condition (*M* = 2.90, *SD* = 0.87 as compared to *M* = 3.01, *SD* = 0.84, *p* = .040, Hedges’ *g* = 0.13), more unrest (*M* = 2.61, *SD* = 0.92 as compared to *M* = 2.76, *SD* = 0.90, *p* = .015, *g* = 0.16), and more stressed (*M* = 1.03, *SD* = 1.07 as compared to *M* = 0.89, *SD* = 1.04, *p* = .031, *g* = −0.14). There was no significant difference on the alertness versus tiredness subscale, *p* = .071. However, as both conditions required participants to stay focused, we expected a smaller difference as compared to the other ratings. There was also a main effect of Stage on all variables (all *p* ≤ .001, 0.103 ≥ η^2^_part_ ≥ 0.034) except for the alertness versus tiredness scale, indicating that the perceived stress level was higher in the application stage than in the baseline stage.

On the task level, there were significant Condition x Stage interaction effects on arousal and valence in all tasks (all *p* ≤ .03, 0.015 ≥ η^2^_part_ ≥ 0.005) except for valence in the follow-the-circle task, *F*(1, 992) = 0.03, *p* =.873, η^2^_part_ = 0.00. Descriptively, there was a greater increase in arousal and negative valence from the baseline stage to the application stage in the high-stress condition as compared to the low-stress condition. Post hoc comparisons of the variables with significant interaction effects revealed no significant between-group differences for any variable in the baseline stage, but in the application stage, participants in the high-stress condition reported a higher arousal and a more negative valence as compared to participants in the low-stress condition (all *p* ≤ .03, 0.206 ≥ *g* ≥ 0.131). Again, there was a main effect of Stage on all variables (all *p* < .001, 0.107 ≥ η^2^_part_ ≥ 0.041) except valence in the follow-the-circle task, indicating that participants felt more aroused and more negative valence after the mouse tasks in the application stage as compared to the baseline stage.

The results support the success of the stress manipulation. The effect sizes were small, but consistent across the measures and time points, indicating that participants felt more stressed during the mouse tasks in the high-stress condition as compared to the low-stress condition.

### Frequentist analysis of the effects of stress on individual mouse features

We used the same mixed ANOVA analysis to test whether the mouse usage features in each task differed between the conditions. To account for the multiple tests in each task, we applied Bonferroni correction to the alpha level of 5%. However, instead of focusing on significance alone, we looked for noticeable mouse features or patterns of interest emerging in a single mouse task or across several mouse tasks.

We tested 16 mouse usage features in the point-and-click task (Bonferroni-corrected *α* = .0031). There were no significant Condition × Stage interaction effects when considering the corrected alpha levels (as in the other sections). Ignoring alpha correction, there were 4 interaction effects at *p* < .05: working time, average speed, average positive acceleration, average negative acceleration. Descriptively, there was a larger decrease in working time from the baseline stage to the application stage in the high-stress condition as compared to the low stress condition (Δ_high-stress_ = −0.51 s, Δ_low-stress_ = −0.23 s), a larger increase in speed (Δ_high-stress_ = 13.24 pixels/s, Δ_low-stress_ = 8.14 pixels/s), a larger increase in positive acceleration (Δ_high-stress_ = 0.35 pixels/s^2^, Δ_low-stress_ = 0.20 pixels/s^2^), and a larger increase in negative acceleration (Δ_high-stress_ = −0.31 pixels/s^2^, Δ_low-stress_ = −0.18 pixels/s^2^). Post hoc comparisons of those variables revealed no significant between-group differences for any variable in either the baseline or application stage.

We tested 16 mouse usage features in the drag-and-drop task (Bonferroni-corrected *α* = .0031). There were no significant Condition × Stage interaction effects when considering the corrected alpha levels. Ignoring alpha correction, there were four interaction effects at *p* < .05: average speed, standard deviation in speed, average positive acceleration, and average negative acceleration. Descriptively, there is a larger increase in the average speed from the baseline stage to the application stage in the high-stress condition as compared to the low stress condition (Δ_high-stress_ = 28.91 pixels/s, Δ_low-stress_ = 21.00 pixels/s), a larger increase in standard deviation in speed (Δ_high-stress_ = 27.18 pixels/s, Δ_low-stress_ = 14.40 pixels/s), a larger increase in positive acceleration (Δ_high-stress_ = 0.58 pixels/s^2^, Δ_low-stress_ = 0.37 pixels/s^2^), and a larger increase in negative acceleration (Δ_high-stress_ = −0.49 pixels/s^2^, Δ_low-stress_ = −0.31 pixels/s^2^). Post hoc comparisons of those variables revealed no significant between-group difference in the baseline stage. There was one significant between-group difference for the average speed in the application stage (*M*_high-stress_ = 457.46 pixels/s, *SD*_high-stress_ = 83.69 as compared to *M*_*l*ow-stress_ = 444.86, *SD*_*l*ow-stress_ = 82.40, *p* = .02, *g* = −0.15).

We tested 13 mouse features in the slider task (Bonferroni-corrected *α* = .0038). There was one significant Condition × Stage interaction effect for the average angle when considering the corrected alpha levels, *F*(1, 983) = 8.634, *p* = .0034, η^2^_part_ = 0.009. Descriptively, there was a greater increase in the angle from the baseline stage to the application stage in the high-stress condition as compared to the low-stress condition (Δ_high-stress_ = 0.20°, Δ_low-stress_ = 0.02°). Post hoc comparison revealed no significant between-group difference for the average angle neither in the baseline stage nor in the application stage. Ignoring alpha correction, there were an additional three interaction effects at *p* < .05: average speed, average positive acceleration, and average negative acceleration. Descriptively, there was a larger increase in speed from the baseline stage to the application stage in the high-stress condition as compared to the low stress condition (Δ_high-stress_ = 23.87 pixels/s, Δ_low-stress_ = 15.84 pixels/s), a larger increase in positive acceleration (Δ_high-stress_ = 0.41 pixels/s^2^, Δ_low-stress_ = 0.25 pixels/s^2^), and a larger increase in negative acceleration (Δ_high-stress_ = −0.36 pixels/s^2^, Δ_low-stress_ = −0.21 pixels/s^2^). Post hoc comparisons of those variables revealed no significant between-group differences for any variable in either the baseline or application stage.

We tested 14 mouse features in the follow-the-circle task (Bonferroni-corrected *α* = .0036). There were no significant Condition × Stage interaction effects when considering the corrected alpha levels, and no interaction effects at when ignoring alpha correction.

Overall, the results do not converge into a clear picture about the effect of stress on mouse usage. Out of the 59 mixed ANOVAs on all variables in all tasks, only the Condition × Stage interaction effect of the average angle in the slider task remained significant after Bonferroni correction. When ignoring Bonferroni correction, there was a pattern of significant interaction effects on average speed as well as average positive and negative acceleration in the point-and-click task, drag-and-drop task, and slider task, hinting that stress increases mouse speed and acceleration. There was also a main effect of Stage for most mouse features in all mouse tasks, indicating a practice effect from the first to the second time a mouse task was performed.

### Machine learning analysis

The principle of supervised machine learning is to use training data to learn a function (or machine learning model) that best maps inputs (e.g., mouse usage features) to outputs (e.g., the stress condition) and then evaluate the goodness of the model on an independent test dataset (James et al., 2013). Note that there are potentially an infinite number of models that can be fitted to the data, and there exists no model that works best for every problem (Wolpert & Macready, 1997). Therefore, the machine approach tests whether a specific model or a specific set of models is better able to map mouse usage to stress than a baseline/null model. A positive test indicates that a systematic relationship between stress and mouse usage may exist, but a negative test does not allow the reverse conclusion, that there exists no systematic relationship between stress and mouse usage, as there could be untested models for which the relationship shows up.

#### Prediction of condition

In the first step of the machine learning analysis, we tried to predict Condition (high-stress versus low-stress) for each mouse task using the mouse usage features as the model input. Prediction performance was assessed with five-fold cross validation and results in the performance evaluation criterion *relative number of correct condition predictions* (i.e., accuracy). To test the significance of the model’s classification performance, it was compared to a distribution of 500 model performance tests on permutated condition labels (permutation test; Ojala & Garriga, 2010). If the model with the true condition labels had a higher accuracy score than the models with the permutated condition labels at least 475 times (*p* < .05), we considered it significantly better than random. All model input features were standardized using a robust standard scaler as implemented in the *sklearn* machine learning package in Python (Version 0.20.1; Pedregosa et al., 2011).

Given the infinite number of possible models, we employed the following strategy to come up with a specific set of models, which we deemed adequate to handle the data and which have been used in similar studies (cf. Can et al., 2019; Yamauchi & Xiao, 2018): (1) We used three common algorithms: logistic regression (LogReg), support vector machine classification (SVC), and random forest classification (RFC). Following the rationale of conservative hypothesis testing (Yamauchi & Xiao, 2018), we used the default model hyper-parameters as provided by the *sklearn* package (Version 0.20.1; Pedregosa et al., 2011) and did not perform hyper-parameter tuning. LogReg has a LIBLINEAR solver, a L2 regularization penalty, and an inverse regularization strength of *C* = 1.0. SVC has a radial basis kernel function, a gamma of 1/(the number of features × the variance of the flattened input feature matrix), and the inverse regularization strength is *C* = 1.0. RFC uses 50 trees and has no maximum tree depth (see code). (2) The baseline stage data (i.e., baseline data) allowed us to consider individual differences in mouse usage. To do so, we calculated the difference scores between the mouse features in the application stage and baseline stage and used said difference scores as the model input features. In an alternative approach, we ignored the baseline data and used the mouse features of the application stage as the model input features instead of the difference scores. The combination of all these options resulted in six classification models (3 algorithms × 2 baseline inclusion approaches) per mouse task. Note that conducting individual permutation significance tests with each model might capitalize on chance. Bonferroni correction decreased the critical *p* value of the permutation test to 0.05/6 = .0083, meaning that the model with the true condition labels must outperform the models with the permutated condition labels at least 496 out of 500 times. Again, instead of looking on significance alone, we looked for noticeable patterns of interest emerging in a single mouse task and across several mouse tasks (Table 2).

**Table 2:** Results of the condition prediction (machine learning classification)

Algorithm	Point-and-click task		Drag-and-drop task		Slider task		Follow-the-circle task	
	Accuracy	*p*	Accuracy	*p*	Accuracy	*p*	Accuracy	*p*
Application stage features (without baseline)								
LogReg	52	0.198	51	0.501	**54**	0.034^*^	50	0.5808
SVC	51	0.411	**54**	0.044^*^	53	0.122	48	0.9441
RFC	48	0.880	53	0.072	53	0.074	46	0.99
Difference score features (with baseline)								
LogReg	53	0.090	53	0.116	**56**	0.002^**^	50	0.7106
SVC	54	0.060	52	0.196	**59**	0.002^**^	51	0.8124
RFC	51	0.222	51	0.287	**53**	0.046^*^	50	0.4671

*Note*. The accuracy columns represent the mean five-fold-cross validation score. The *p* columns represent the *p* values of the permutation tests. LogReg: logistic regression; SVC: support vector machine classification; RFC: random forest classification. *p < .05, **p < .0083 (Bonferroni-corrected *p* value)

In the point-and-click task, no model significantly predicted the stress condition better than random for both, the Bonferroni-corrected critical *p* value and the uncorrected critical *p* value of .05.

In the drag-and-drop task, no model significantly outperformed the Bonferroni-corrected critical *p* value of the permutation test. Ignoring Bonferroni correction, there was one model with a significant prediction performance: the SVC when ignoring the baseline (54% accuracy, *p* = .04).

In the slider task, two models significantly outperformed the Bonferroni-corrected critical *p* value of the permutation test: the LogReg (56% accuracy, *p* = .002) and the SVC (59% accuracy, *p* = .002) when considering the baseline. Ignoring Bonferroni correction, there were two additional models with significant prediction performance: the LogReg when ignoring the baseline (54% accuracy, *p* = .04) as well as the RFC when considering the baseline (53% accuracy, *p* = .05).

In the follow-the-circle task, no model significantly predicted the stress condition better than random either with Bonferroni-corrected critical *p* value or with an uncorrected critical *p* value of .05.

In sum, the results of the condition classification do not converge to a clear picture about an effect of stress on mouse usage. Out of 24 prediction classifications, two remained significant after correcting for multiple testing. Just as in the frequentist analysis, the significant results emerged in the slider task. When ignoring Bonferroni correction, 5 out of the 24 predictions were significant (i.e., four in the slider task and one in the drag-and-drop task). Overall, all accuracy scores were close to random guessing with 59% accuracy as the best result.

#### Predicting valence and arousal rating

Our experimental design assumed the existence of two groups with dichotomous stress levels (high versus low). In reality, stress is continuous, and the subjective nature of stress causes the stress manipulation to have different effects on participants independent of experimental condition. To account for this, we additionally collapsed the group design and performed correlative analyses independent of experimental conditions, to analyze the relationship between stress and mouse usage on an individual level. Specifically, we used the mouse usage data to predict the valence and arousal ratings of each task (regression analysis). Contrary to the condition classification, this correlative approach does not allow for a causal interpretation of the stress manipulation on mouse usage.

The regression analysis followed similar steps as the condition classification. We used five-fold cross validation to evaluate our model’s performance. The performance evaluation criterion of the regression model was the coefficient of determination (*R*^2^). A null model, which always predicts the mean value of the outcome variable (arousal or valence rating), disregarding the input features (mouse usage features), has a *R*^2^ score of 0; thus a correlation between valence or arousal and mouse usage is represented by models with *R*^2^ > 0. The significance of a regression model with *R*^2^ > 0 was tested by comparing it to a distribution of 500 model performance tests on permutated valence/arousal scores. If the model with the true valence/arousal ratings had a higher *R*^2^ than the models with permutated valence/arousal ratings for at least 475 times (p < .05), we considered it better than random. All model input features were standardized using *sklearn’s* robust standard scaler (Version 0.20.1; Pedregosa et al., 2011).

Again, we tested a set of different models: (1) We used three common algorithms with default hyper-parameters as provided by the *sklearn* package (Version 0.20.1; Pedregosa et al., 2011) and did not perform hyper-parameter tuning: linear regression (LinReg), support vector machine regression (SVR), and random forest regression (RFR). LinReg is an ordinary least-squares regression, and the model included an intercept. SVR has a radial basis kernel function, a gamma of 1/(the number of features × the variance of the flattened input feature matrix), and the inverse regularization strength is *C* = 1.0. RFR has 50 trees and there is no set maximum tree depth (see code). (2) We controlled for the baseline by using the difference scores of the mouse features between the application stage and baseline stage as the model input and the difference scores of the valence and arousal ratings as the dependent variable (versus ignoring the baseline and using the mouse usage features as well as the valence/arousal ratings of the application stage). Overall, the combination of all options resulted in six regression models (3 algorithms × 2 baseline inclusion approaches) per mouse task, per dependent variable (valence, arousal). Bonferroni-corrected *p* values of the permutation tests were 0.05/6 = .0083.

No model achieved a *R*^2^ considerably larger than 0, omitting the need for permutation tests (Table 3). To this effect, we found no correlation between mouse usage and self-reported arousal or valence.

**Table 3:** Results of the valence and arousal predictions (machine learning regression)

Algorithm	Point-and-click task		Drag-and-drop task		Slider task		Follow-the-circle task	
	Valence *R*^*2*^ score	Arousal R^2^ score	Valence *R*^*2*^ score	Arousal R^2^ score	Valence *R*^*2*^ score	Arousal R^2^ score	Valence *R*^*2*^ score	Arousal *R*^*2*^ score
Application stage mouse features and valence/arousal ratings (without baseline)								
LinReg	−0.01	−0.04	−0.00	−0.03	−0.01	0.00	−0.00	−0.03
SVR	−0.12	−0.08	−0.03	−0.08	−0.07	−0.05	−0.10	−0.05
RFR	−0.05	−0.06	−0.05	−0.05	−0.04	−0.08	−0.09	−0.07
Difference score mouse features and valence/arousal ratings (with baseline)								
LinReg	−0.01	−0.03	−0.00	−0.01	0.00	−0.02	−0.02	−0.02
SVR	−0.08	−0.06	−0.06	−0.06	−0.10	−0.06	−0.09	−0.04
RFR	−0.07	−0.02	−0.06	−0.02	−0.05	−0.04	−0.02	−0.05

*Note*. The *R*^*2*^ score columns represent the mean five-fold-cross validation *R*^*2*^ scores. LinReg: linear regression; SVR: support vector machine regression; RFR: random forest regression

#### Alternative machine learning approaches

**Fig. 7:**
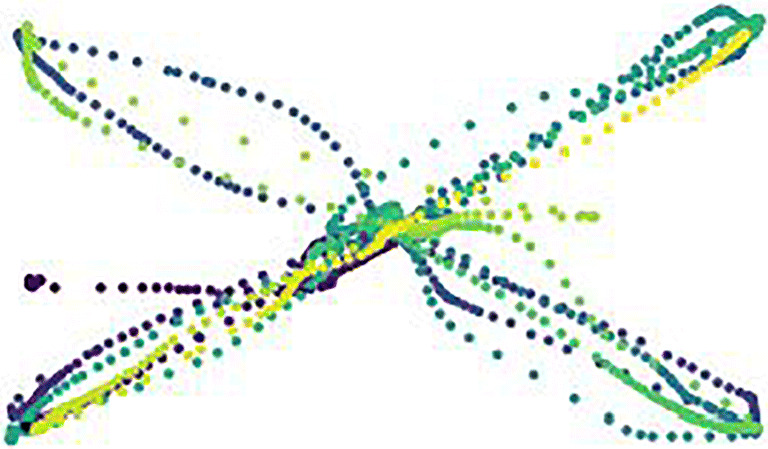
Visualization of the mouse usage behavior of a sample participant in the drag-and-drop task. The rectangular frame represents the computer screen. The dots represent single mouse data points. Mouse movement data points are chronologically ordered from purple to yellow. Mouse clicks are represented by black dots

To avoid the potential shortcoming of having selected a specific set of mouse usage features from an infinite feature space accompanied by possible information loss—as done in the previous approach—we implemented an additional explorative machine learning approach, which used the raw mouse usage data as the model input: We created images of the mouse usage during each task and used them as the model input data to predict the stress condition (classification) and the self-reported valence/arousal scores (regression). The images were scatter plots of the mouse data point’s *x*- and *y*-coordinates with the *x*- and *y*-axis of the plot corresponding to the participant’s screen. Here, we used the original mouse usage data points instead of the interpolated data points. To add temporal information to the plots, the sequence of the mouse movement data was visualized by assigning each consecutive data point a unique color, which matched a predefined color pattern from purple (first data point) to yellow (last data point). The mapping of data point to color was relative to the total number of data points, meaning that an increase in data points increased the intermediate color steps between purple and yellow, but the first data point always had the same purple color and the last data point always had the same yellow color. To separate mouse click data points from mouse movement data points, mouse click data points are black (Fig. 7).

The algorithm to analyze the image data was a convolutional neural network (resnet 34, as implemented by the *fastai* package [version 1.0.59, 2019] in Python). We controlled for baseline by merging the images of the mouse usage during the application stage and baseline stage into a single image by stacking them onto each other. In an alternative approach, we only used the application stage images as the model input. This resulted in two models (1 algorithm × 2 baseline inclusion approaches) per mouse task per prediction (condition classification, regression on valence, regression on arousal). Because the image analysis approach was exploratory and computationally expensive, we tested the prediction performance with a single randomly drawn train (80% of the data)-test (20% of the data) split instead of five-fold cross-validation and did not perform permutation tests.

The results of the machine learning approach using the mouse usage images are presented in Table 4. There are no noticeable differences to the results of the machine-learning approach using the selected set of mouse usage features. The best classification accuracy was 58% in the slider task when ignoring the baseline. In the regression, we found no correlation between mouse usage and self-reported arousal or valence (all *R*^*2*^ < 0).

**Table 4:** Results of the condition (classification), valence, and arousal predictions (regression) using the mouse usage images as the model input

	Included only the data of the application stage (without baseline)			Included the data of the application stage and baseline stage (with baseline)		
Mouse task	Condition prediction (Accuracy)	Arousal prediction (R^2^ score)	Valence prediction (R^2^ score)	Condition prediction (Accuracy)	Arousal prediction (R^2^ score)	Valence prediction (R^2^ score)
Point-and-click task	53	−1.27	−4.23	44	−1.55	−4.41
Drag-and-drop task	54	−1.10	−5.22	56	−1.24	−2.64
Slider task	58	−1.56	−4.66	50	−2.63	−2.93
Follow-the-circle task	49	−1.22	−2.74	55	−2.10	−2.14

*Note*. The algorithm was a convolutional neural network (resnet 34). The model that was trained with 80% of the sample drawn at random, and the results represent the prediction performance on the remaining 20% of the sample

Because all results are fluctuating around the random guess mark, it is hard to judge the feasibility of the image analysis approach. We therefore additionally tested whether the approach is able to correctly classify between two mouse tasks (point-and-click task versus drag-and-drop task) as they should be separable. The accuracy of the image classification approach was 100%. We likewise tested the accuracy of the mouse features approach (here, we exemplarily used a support vector machine classifier and ignored the baseline). The accuracy was 100%, as well.

## Discussion

The computer mouse is a ubiquitous sensor that captures dynamic real-time data about human behavior at millisecond precision (Hehman et al., 2015). These qualities have been recognized by a growing number of researchers in cognitive science that utilize computer mouse tracking as research methodology to better uncover the processes of how we think (Freeman, 2018; Stillman et al., 2018). The present paper combined mouse tracking and affective computing research to investigate whether the computer mouse would prove an effective research methodology to better uncover the processes of how we feel. Specifically, we aimed to explore the relationship between stress and mouse usage during goal-directed tasks. To do so, we conducted an online experiment in which participants worked on four different mouse usage tasks (a point-and-click task, drag-and-drop task, slider task, and follow-the-circle task) in either a high- or low-stress condition. We used a wide range of statistical approaches to find patterns in the mouse usage data that are linked to stress.

While the manipulation check revealed that participants in the high-stress condition reported a small but consistently higher stress level on different self-report measures compared to participants in the low-stress condition, we found no clear relationship between stress and mouse usage behavior. There was some very tentative statistically significant evidence as to an effect of stress on mouse usage in the slider task with both frequentist and machine learning analysis. Additionally, there was a tentative pattern of a stress-related increase in participant’s cursor speed and acceleration for the point-and-click task, the drag-and-drop task and the slider task. However, all effect sizes were marginally small (all η^2^_part_ < .01, which is the cut-off for a small effect according to Cohen, 1988). All condition classifications were close to random guessing (a maximum accuracy of 59% is not reliable for stress detection), and there was no correlative relationship between mouse usage and participants valence and arousal ratings in any mouse task. With a sample of *N* = 994, the probability of missing a substantial effect of stress on mouse usage was low, although the small observed effect of the stress manipulation potentially set an upper limit on the expected effect of stress on mouse usage. Considering the effect on perceived stress (η^2^_part_ = .015) as the expected effect size, the power to find such an effect of stress on mouse usage was .82 for the Bonferroni-corrected alpha level (.0031) and .97 for the uncorrected alpha level (calculated with G*Power; version 3.1.9.7.; Faul et al., 2007). With the machine learning analyses, we did not observe major fluctuations between the prediction results of different models or within different folds of the five-fold cross validation, indicating that the sample was large enough for the prediction models to be stable and therefore reliable.

In sum, the results provide little evidence for a meaningful relationship between stress and mouse usage. Nonetheless, it is important to note that the exploratory nature of the data analysis does not rule out the existence of a systematic relationship between mouse usage and stress, which might be harder to find than we were able to in the present study with the given dataset, the given stress manipulation, and the given data analytical approaches.

### Placing the results in the context of the existing literature

The present study’s findings align with the hitherto mixed evidence on the relationship between mouse usage and affective states of previous studies. Although individually, most studies drew a more positive conclusion from their results, collectively, the high number of apparently abandoned pilot projects paired with heterogeneous and hard-to-integrate empirical evidence strengthens our finding of a lack of a systematic relationship between mouse usage and stress. We argue that a similar conclusion can also be drawn from the—to our knowledge—most comprehensive study published in the research area so far (Yamauchi & Xiao, 2018). The authors interpret their results as promising and in favor of an effect of affective states on mouse usage, but they found a mix of both significant and non-significant correlations between mouse usage and specific affective states for specific samples (men or women) in specific experimental settings. In Study 1 (no emotion manipulation), they found correlations between mouse usage and anxiety in men and women. In Study 2 (music-based emotional manipulation), they found correlations with positive emotions (positive affect, joviality, self-assurance, attentiveness), but not with negative emotions (negative affect, sadness, fear, hostility) in men and women. In Study 3 (film-based emotion manipulation), they found correlations between mouse usage and positive affect and attentiveness in women and correlations between mouse usage and self-assurance in men (participants rated the same emotions as in Study 2). In Study 4 (picture-based emotion manipulation), they found correlations between mouse usage and self-rated valence and arousal in men and women. The overall pattern in the results is not interpretable in a straightforward way. Moreover, the authors did not adjust the *p* values of their models’ significant tests to the fact that they used different algorithms, iterated over several dependent variables, and split the sample into subsamples. As regards the ecological validity of their results, the study included only laboratory experiments and a sample predominantly comprising students, possibly overestimating a general effect of affective states on mouse usage when taking hardware variance and interpersonal variance into account. Considering the studies about the relationship between mouse usage and stress, Freihaut and Göritz (2021) similarly found no systematic effect of stress on mouse usage. Sun et al. (2014) showed mixed evidence for an effect of stress on mouse usage. The results of Kowatsch et al. (2017a) more consistently pointed towards an effect of stress on mouse usage, but were based on a very small sample of *N* = 19, and the same authors (2017b) found no clear correlation between mouse speed and self-rated valence and arousal in their field study.

Taken together, the results of the present study and the ambiguous empirical state-of-the-art favor a more pessimistic or at least cautious view of a systematic effect of affective states on mouse usage than previous research might suggest. More importantly, the combined results highlight that the research area lacks a theoretical foundation and faces tremendous methodological challenges. On the one hand, both aspects hinder the formulation of research hypotheses and complicate the integration of different results, while on the other hand, they promote finding and reporting unreliable outcomes, as the data analyses approaches are not standardized and exploratory.

### Using mouse tracking for stress measurement and research

Affective states, stress, and sensorimotor behavior are complex and dynamic phenomena by themselves (Gallivan et al., 2018; McEwen, 2000; Russell, 2003). Accordingly, their interplay is unlikely to be less complex. In the introduction, we presented evidence from different research areas about effects of stress on cognitive and biomechanical processes potentially involved in mouse usage, but the results of the present study demonstrate that putting the empirical and theoretical pieces together is impossible for the time being.

From a stress measurement perspective, the results are sobering. Even if the sparse evidence in favor of an effect of stress on mouse usage holds true in further research, the effects are likely too small to reliably infer stress from cross-situational mouse usage. A theoretical interpretation of the empirical evidence suggests that mouse usage behavior represents the end result of complex and context specific processes, which can hardly be translated into a generalized stress marker similar to other markers that target more automated and uniform stress-related processes such as an increase in heart rate resulting from the activation of the sympathetic nervous system (Pruessner et al., 2010). The present study and most other studies in the research area, however, neglected the major advantage of the computer mouse as a continuous and individualized data collection tool. Future research on stress measurement via the computer mouse should therefore focus on such an individualized and long-term approach. It might be possible to carve out mouse usage patterns that relate to stress or other affective states from data captured from the same individual over multiple times and tasks. To our best knowledge, such research hardly exists. Kowatsch et al. (2017b) collected mouse usage data from office workers over multiple weeks, but only published preliminary analyses that showed mixed results. Khan et al. (2013) collected longitudinal data from everyday computer interactions, including mouse usage events, and found correlations between computer interactions and self-rated valence and arousal at an individual level. Finally, Pimenta et al. (2016) captured mouse usage during classwork in a computer laboratory and found that participants with higher fatigue levels showed more variance in their mouse usage. They were able to predict self-rated fatigue levels from the mouse data of participants in a test set at 81% accuracy. More generally, putting the focus on long-term tracking of individualized stress levels has potential for a better understanding of the dynamics of stress and the relationship between stress and the development of affective disorders such as chronic stress and burnout (Adjerid & Kelley, 2018).

From a theory-building perspective, the results highlight a need for systematic research on the interplay between stress and sensorimotor behavior. To this effect, mouse tracking might be a promising research methodology (similar to the use of mouse tracking in cognitive science). We exemplarily propose potential topics of interest which emerged from theoretical reflections about the meager results of the study at hand:

Our definition of stress postulates that a (dis-)stress reaction requires a perceived and threatening discrepancy between situational demands and coping resources (Zapf & Semmer, 2004). This definition implicates the existence of a threshold beyond which the individual feels distressed. Common measurement approaches of stress (such as the questionnaires used in the present study), however, do not incorporate such a threshold. An increase in physiological arousal paired with an increase in negative valence is commonly interpreted as an increase in the stress level independent of their absolute values. Given the existence of a threshold, mouse usage might not be linearly related to arousal and valence, but might only change when the threshold is surpassed. Moreover, this surpassing of the threshold might not be amenable to self-insight (yet), but merely register in physiological or other non-conscious parameters, whereas becoming aware of one’s own feeling of distress might require surpassing another threshold or the presence of additional external or internal characteristics. On a similar note, different cognitive, emotional, physiological and behavioral processes related to the stress reaction might differ in sensitivity towards situational demands and therefore have measurable onsets at different intensity levels. This hypothesis is backed by research showing that self-report and physiological stress markers do not necessarily correlate with each other (Liapis et al., 2015; Stalder et al., 2017). Future research could therefore try to simultaneously measure and compare different stress measurement approaches (e.g., self-report, physiological and behavioral measures) at varying stress levels and at qualitatively different sources of stress.

The goal-directed mouse tasks in the present study as in most other studies were directly or indirectly tied to performance (e.g., clicking on targets as fast as possible). According to Yerkes-Dodson law (Yerkes & Dodson, 1908), there is an inverted quadratic relationship between performance and arousal, which is backed by research on the effects of stress on memory performance (Lupien et al., 2007). In terms of motor performance, van Galen and van Huygevoort (2000) argue that detrimental effects of stress can be biomechanically compensated to some degree. Similarly, according to sparse capacity models, humans are temporarily able to compensate for a decrease in performance during an increased workload or stress by resorting on spare resources and an increase in effort (Casali & Wierwille, 1983; Hockey, 1997; Pimenta et al., 2016). Likewise, a meta-analysis about the relationship between stress-related anxiety and sports performance only showed weak correlations (Craft et al., 2003). Again, this indicates that stress and mouse usage might not be related in a straightforward manner in mouse performance oriented-tasks and, more generally, that behavioral stress measures and self-report or physiological stress measures do not necessarily share the same response pattern to an increase in the stress level.

Lastly, the results of the present study raise the question of how the task affects the relationship between stress and mouse usage, as there was slight evidence about an effect of stress on mouse usage in the slider task, but less evidence in the point-and-click as well as in the drag-and-drop task and no evidence in the follow-the-circle task. This might indicate that there is no cross-situational or task-independent direct effect of stress on mouse usage, but—if any—an effect of stress on task processing, which in turn is reflected in mouse usage. To give an example, Kowatsch et al. (2017a) argued that stress increases noise in the sensorimotor process, which causes an increase of stutter or micro-deviations in mouse movements. Such an effect represents a direct effect of stress on mouse usage, which should be visible across different tasks. In contrast, Hibbeln et al. (2017) drew on Attentional Control theory (Eysenck et al., 2007) and postulated that negative valence causes a shift in attentional focus from goal orientation to stimulus orientation resulting in a different processing of information during a goal-directed task. Such an effect represents an indirect effect of stress on mouse usage, because stress changes task execution (e.g., the decision between alternative stimuli), which is then mirrored in changes in mouse usage. Furthermore, the relationship between stress and mouse usage might also depend on the task demands. Neurologically, stress causes a shift in activation of brain regions responsible for higher-order cognitive processes in favor of brain regions responsible for more immediately adaptive and habitual responses (Arnsten, 2009), that is, the activation of adaptive processes that prepare to fight-or-flight a threatening situation. Therefore, simple tasks might be affected differently from tasks with higher demands on cognitive or motor skills. Accordingly, the shape of the Yerkes-Dodson curve depends on the task difficulty and performance in simple tasks and does not necessarily follow a quadratic function, but can plateau with increasing arousal (Diamond et al., 2007). Future research should therefore test the effects of varying stress levels on mouse tasks with varying cognitive and motor demands. As the motor demands during regular computer usage most likely do not exceed the motor demands of the mouse tasks in the present study, we suggest to put an emphasis on the development and testing of mouse tasks with varying cognitive demands. Examples for such tasks are the puzzle-solving task as used by Hibbeln et al. (2017) or the decision-making task as used by Yamauchi and Xiao (2017).

### Methodological considerations of the study

Although the manipulation check revealed a higher stress level in the high-stress condition than in the low-stress condition consistently across different self-report measures, all effect sizes of the difference in stress were small. As such, the stress manipulation might have been successful but too weak to have had a distinct effect on mouse usage. To circumvent the small group differences and to accommodate the subjective nature of stress, we additionally performed correlative analysis between mouse usage and participants’ self-rated valence and arousal, which did not reveal any meaningful relationship in any mouse task.

The present study’s stress manipulation protocol relied on a hard (versus easy) counting task and a threatening (versus neutral) framing to induce a high (versus low) stress level between the conditions. The counting task is a novel task and therefore not (yet) well established. We developed the counting task because of a lack of stress manipulation tasks that fit the needs in our experiment. In contrast to other established computerized stressor tasks such as the Stroop task (Stroop, 1935), the counting task is easy to understand and requires little practice. Moreover, the counting task does not require mouse usage, which otherwise could have affected the succeeding mouse tasks, and its difficulty can adjusted by changing the number of targets and distractors. We decided against a mental arithmetics task as another typical stress manipulation task (Dickerson & Kemeny, 2004), which has similar characteristics, because counting squares is a more neutral task than solving mathematical equations and therefore might have a more homogenous effect across participants. The small difference between conditions as well as the main effect of the experimental stage with higher stress levels in the application stage than in the baseline stage indicates that the counting task (in addition to the framing) can be used as a new stress manipulation protocol, but also highlights the need for adjustments to induce more distinct stress levels between the conditions. Potential adjustments to the counting task include changes of the number of targets and distractors or changes in the time limit. The task might also profit from including additional shapes, colors (e.g., counting the blue squares only) or more complicated instructions such as counting the difference between blue and green squares to add a mental arithmetic component. Lastly, it is also possible to dynamize its difficulty based on individual performance.

The framing was added to the stress manipulation protocol to include a social evaluative threat. In an online setting, it was not possible to add a real-time social interaction to the stress manipulation protocol. We therefore targeted at a more self-evaluative threat by framing the application stage in the high-stress condition as an intelligence test. This framing might have resulted in a weaker stress reaction than a social evaluative situation in which participants have to perform in front of an audience such as in the Trier Social Stress Test (Kirschbaum et al., 1993). The Montreal Imaging Stress Test (Dedovic et al., 2005) includes a fictional performance criterion about the expected and average performance in a mental arithmetic task to include a social-evaluative threatening component, which might be stronger than simply stating that the task represents a performance test. However, we decided against the inclusion of a more explicit performance pressure framing, because some participants who are not able to remotely meet the performance criterion might be discouraged rather than stressed, or they might realize that the task is make-believe.

Lastly, the high degree of standardization of the stress manipulation protocol might have caused the conditions to be too similar to have achieved a large difference in stress level. We chose to maximize the comparability of the two stress conditions and thus internal validity. However, in the interest of creating unequal stress levels between the conditions, it might be reasonable for future research to sacrifice some of the standardization in favor of the intensity of the stress manipulation. For example, Sun et al. (2014) chose such an approach and let participants do mindfulness meditation in the low stress condition versus mental calculations under time pressure in a social-evaluative situation in the high stress condition.

We exhaustively tried to find correlations between mouse usage in stress in our data using multiple statistical approaches, which can be considered a strong point of the study. We showed that these data analytical procedures were unable to find a systematic relationship between stress and mouse usage in the data, but it might be possible that other approaches are able to do so. To the best of our knowledge, we covered the core approaches used in other studies in this research area. Feasibility tests (i.e., classifying the point-and-click versus the drag-and-drop task) showed that the machine learning models were able to handle the mouse usage data, thus justifying their use. Moreover, we introduced a novel image based approach to analyze mouse usage data without the need to dissect the raw data into features. The approach is exploratory and its implementation might be improved with more domain expertise. More generally, transforming the mouse data into images might encourage others to rethink how their data can be analyzed in novel ways.

Finally, we consider the experimental design of this study and its large and heterogeneous sample to be strong points of this research.

## Conclusion

Computer mouse tracking offers a simple, unobtrusive, and cost-efficient way to gather continuous behavioral data, which might contain useful information for different fields of psychological science. The present study tested the feasibility of utilizing computer mouse tracking to measure people’s individual stress levels. The results do not show a clear relationship between stress and mouse usage. One the one hand, this suggests that generalized stress measurement via the computer mouse is likely not feasible. On the other hand, it highlights the need for theoretical advancements about the interplay between stress and sensorimotor behavior. In line with the open science movement (Crüwell et al., 2018), we bring forward the sparse and ambiguous findings of this study as well as our research materials in the hopes of fostering a critical discussion and the development of new ideas and approaches.
